# Physiotherapy-Led Musculoskeletal Telephone Triage and Advice Service: A Valid Option for Patients Referred From the Emergency Department

**DOI:** 10.7759/cureus.34720

**Published:** 2023-02-07

**Authors:** Richard J Dowell, Janki Dattani, Mohamed Nagy, Ahmed Al Wadiya, Moustafa Maher, Neil Ashwood

**Affiliations:** 1 Emergency Department, University Hospitals of Derby and Burton NHS Foundation Trust, Derby, GBR; 2 Physiotherapy Department, University Hospitals of Derby and Burton NHS Foundation Trust, Derby, GBR; 3 Trauma and Orthopaedics Department, Cairo University Hospitals, Kasr Alainy, Cairo, EGY; 4 Trauma and Orthopaedics Department, Manchester University Hospitals NHS Foundation Trust, Manchester, GBR; 5 Trauma and Orthopaedics Department, University Hospitals of Derby and Burton NHS Foundation Trust, Derby, GBR; 6 UC Health Orthopaedic Surgery Department, Foot and Ankle Surgery Division, University of Colorado Hospital, Colorado, USA; 7 Research Institute, University of Wolverhampton, Wolverhampton, GBR

**Keywords:** face-to-face appointment, acute injuries, telephone triage and advice service, musculoskeletal, physiotherapy

## Abstract

Introduction

Musculoskeletal (MSK) conditions create a significant demand for healthcare services in the United Kingdom. The emergency department is one of the main providers of initial care for patients with MSK disorders or injuries. As attendances increase within the emergency department the demand for MSK physiotherapy services also increases. The MSK physiotherapy department at Queens Hospital, Burton, GBR introduced a telephone triage and advice (TTAD) service for patients referred from the emergency department to try and reduce waiting times and the number of initial appointments not attended.

The primary outcome of the study was to investigate the number of patients discharged via the TTAD service. Secondly, the study aimed to assess if the TTAD service eased the pressures of face-to-face appointments as well as analyze the effects on the number of failed attendances and canceled appointments for both initial and follow-up face-to-face appointments.

Method

Data were collected retrospectively from the electronic medical records system Meditech Version 6 (Medical Information Technology, Inc., MA) from the months of August, September, and October in 2017 (pre-TTAD) and 2018 (post-TTAD). Once the data had been collected, analysis was performed comparing results from 2017 to 2018 using Statistical Product and Service Solutions (SPSS) (IBM SPSS Statistics for Windows, Armonk, NY) analysis software.

Results

The overall number of referrals from emergency to MSK physiotherapy increased by 11.2% between 2017 and 2018. Following the introduction of the TTAD service, 59.8% of the total referrals were offered a face-to-face initial appointment with 40.2% of patients referred being discharged via the TTAD service in 2018. The percentage of patients that failed to attend the initial appointment in 2018 also fell by 4.9%.

Conclusion

The introduction of a TTAD service for referrals from the emergency department has been demonstrated to be effective in reducing the number of face-to-face appointments required in the MSK physiotherapy management of these patients. Both initial and follow-up face-to-face appointments were lower in 2018 when compared to 2017, this is despite an 11.2% increase in the number of referrals throughout August, September, and October. It can therefore be concluded that the TTAD service also had a positive impact on the failed attendance rate of initial face-to-face appointments.

## Introduction

Musculoskeletal (MSK) conditions are a growing concern worldwide with approximately 1.71 billion people suffering from MSK conditions globally [1]. These conditions create a significant demand for healthcare services with an estimated 9.6 million adults and 12 thousand of children suffering from MSK disorders in the United Kingdom (UK) [2]. The management of MSK conditions can be treated by multiple healthcare provider pathways. In the UK, these can include attendance at the GP, consulting clinics, 111 services, and the emergency department.

The emergency department is one of the main providers of care for MSK disorders in the UK. It has been suggested 3 million from 23.5 million attendances in 2016 were because of MSK conditions [3]. These statistics potentially could be greater, as a further 3 million attendances were reported in this study but were not given a classified diagnosis [3]. It has also been demonstrated the number of emergency department attendances has risen, further increasing the demands on healthcare services. In the UK, it has been reported attendance has increased from 23.5 million in 2016 to 24.5 million in 2018, illustrating an increase of 4% over two years [4].

Following emergency department attendance, many patients with an MSK disorder are managed with a conservative management plan and are referred to MSK physiotherapy services. As a result, this can place an increased demand on physiotherapy services, which can in turn increase waiting times in the physiotherapy outpatient department. In some geographical locations, the waiting lists have exceeded 30 weeks [5]. Long delays to receiving treatment can be detrimental to a patient’s recovery [6], leading to poorer outcomes, thus leading to an increased burden in the healthcare systems for patients with MSK conditions [7,8]. Another consequence of longer waiting times in receiving physiotherapy is the increased number of appointments not attended in the physiotherapy department, accumulating additional time and financial pressures on the healthcare service [9].

To find a solution to meet the demands of the MSK Physiotherapy service, many MSK outpatients’ physiotherapy providers have implemented a telephone triage and advice (TTAD) service. These services are aimed to decrease waiting times and reduce non-attendances [10]. Existing literature investigating the effectiveness of telephone consultations in physiotherapy has been limited [11]. The majority of the literature has analyzed telephone consultations delivered by nursing staff in an Asthma clinic [12]. These studies have been demonstrated to be safe clinically, cost-effective, and widely accepted by users [12,13].

Although there is limited literature in physiotherapy outpatient settings, it has been demonstrated, TTAD assessments can reduce waiting times, whilst being clinically effective in comparison to face-to-face assessments in a physiotherapy department setting [6,11]. From further analysis of the literature, it was apparent that only one study demonstrated the implementation of an option for telephone assessment service in MSK outpatient physiotherapy for patients referred from the emergency department [11]. This study analyzed face-to-face routine appointments in comparison to telephone consultations. The researchers for the study utilized broad inclusion and exclusion criteria to assess the effectiveness of telehealth on multiple patient populations and conditions. A senior physiotherapist was utilized to provide self-management and advice for each of the conditions. Although the study used a small sample size, the researchers concluded the service reduced waiting times and was accepted by service users [11].

At the Queens Hospital Burton site of the University Hospitals of Derby and Burton National Health Service (NHS), the MSK physiotherapy department observed a high rate of failed attendances from patients referred from the emergency department, recording 14.4% of initial appointments not being attended by patients. These findings were not uncommon and were similar to the aforementioned studies [6,10,11].

In response to the difficulty with MSK appointments at Queens Hospital Burton, the outpatient department introduced a TTAD service to contact patients in a timely and more efficient manner. This project was completed to measure the impact of implementing a TTAD service for patients referred to the emergency department. The primary outcome was to investigate the number of patients discharged via the TTAD service. Secondly, aimed to assess if the TTAD service eased the pressures of face-to-face appointments as well as analyze the effects on the number of failed attendances and canceled appointments for both initial and follow-up face-to-face appointments.

## Materials and methods

Data were collected retrospectively from the electronic medical records system Meditech Version 6 (Medical Information Technology, Inc., MA) from the months of August, September, and October in 2017 and 2018. Data collected in 2017 represents the outcome measures before the implementation of the TTAD service and in 2018, post-implementation of the TTAD service. The study was registered under the NHS trust of the University Hospitals of Derby and Burton. Once the data had been collected, analysis was performed comparing results from 2017 to 2018 using Statistical Product and Service Solutions (SPSS) (IBM SPSS Statistics for Windows, Armonk, NY) analysis software.

The collected and analyzed data were categorized into diagnosis (2018 only), face-to-face appointment made, attended initial appointment, canceled initial appointment, failed to attend the initial appointment, treatment completed, canceled follow-up appointment, failed to attend a follow-up appointment, no follow-up appointment required, and finally discharged TTAD service.

Telephone triage pathway

The emergency department completed a paper referral form for every patient requiring physiotherapy follow-up post-discharge from the department. The Queens Hospital Burton site is one of the hospitals employing emergency physiotherapy practitioners (EPP) in the emergency department. The role of the EPP in the emergency department is to assess and treat minor MSK injuries and has been shown to be as effective as other practitioners with a below-national-average re-attendance rate [14]. The EPP prioritizes the referrals from the emergency department and categorizes them for the TTAD service or to receive a face-to-face appointment. The criteria for face-to-face appointment at the point of prioritization was based on the complexity or chronicity of the diagnosis as these groups of patients were deemed to require a higher level of input than those suffering acute minor MSK injuries.

Patients prioritized for the TTAD service were contacted within two weeks of referral. Patients were discharged from the TTAD service if on contact were found to be self-managing or the problem had resolved, if improving but not ready for discharge the patient continued with a telephone review. The criteria for face-to-face appointment at the point of the TTAD were patients who were not improving despite initial advice or if any doubt existed regarding the diagnosis or nature of the injury. For those patients going on to receive a face-to-face appointment, initial advice was given, and further details were collected to better inform the allocation and grade of physiotherapist appropriate for the patient.

## Results

Through the months of August, September, and October, the number of referrals received for MSK physiotherapy from the emergency department increased from 250 in 2017 to 348 in 2018 (Table 1), an increase of 11.2%.

**Table 1:** Referrals and appointments made in 2017 vs 2018 TTAD: telephone triage and advice

Year	2017	2018
Number of Referrals	250	348
Contacted via TTAD	0 (0.0%)	215
Discharged via TTAD	0 (0.0%)	140 (40.2%)
Initial face-to-face	250 (100%)	208 (59.8%)

**Figure 1:**
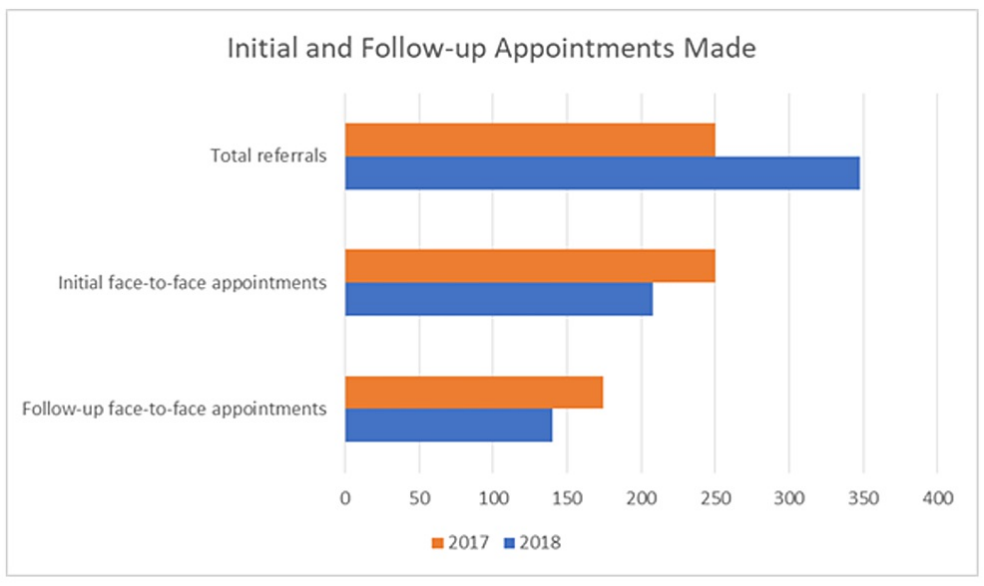
Number of initial and follow-up face-to-face appointments made

Of the 348 patients referred in 2018, 215 were contacted via the telephone triage service with 140 being discharged via the service and 75 being offered a face-to-face appointment (Table 1). The total number of initial face-to-face appointments made reduced in 2018 to 208 compared to 250 in 2017 (Figure 1).

From the initial face-to-face appointments made, 172 were attended in 2017 and 140 in 2018 with 42 being canceled in 2017 compared to 35 in 2018. The number of initial appointments patients failed to attend decreased from 36 (14.4% of total referrals) in 2017 to 33 (9.5% of total referrals) in 2018 (Table 2).

**Table 2:** Outcome of an initial face-to-face appointment

Year	2017	2018
Number of Referrals	250	348
Attended	172 (68.8%)	140 (40.2%)
Canceled	42 (16.8%)	35 (10.1%)
Failed to attend	36 (14.4%)	33 (9.5%)

**Figure 2:**
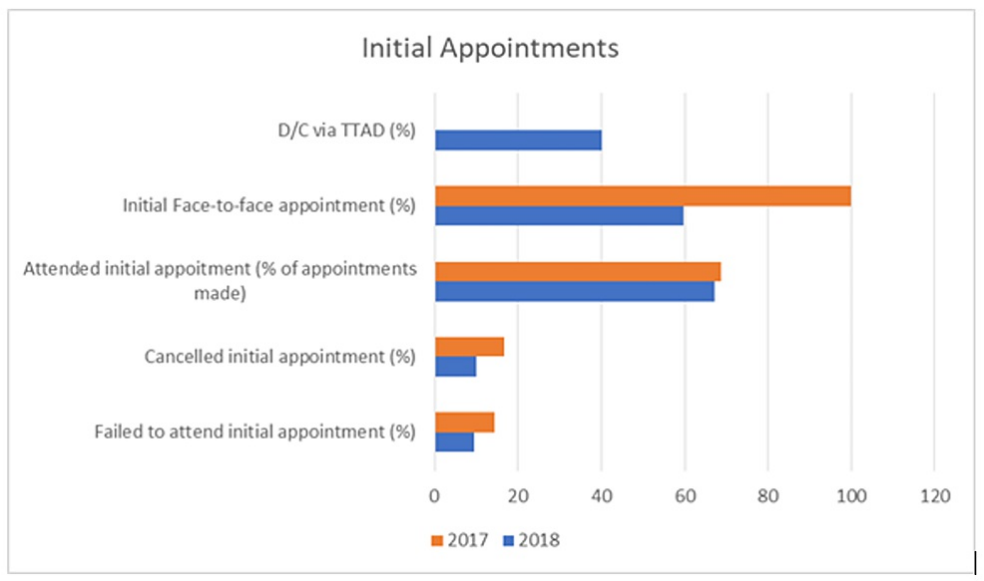
Percentage of patients discharged via TTAD and initial appointment outcomes D/C: discharge, TTAD: telephone triage and advice

A total of 174 patients required a face-to-face follow-up appointment in 2017 compared to 140 in 2018 (Figure 1). The number of patients that completed treatment was 122 in 2017 and 91 in 2018. Canceled appointments were reduced in 2018 to 19 from 22 in 2017 with appointments not attended also decreasing to 30 in 2018 from 40 in 2017 (Figure 2) (Table 3).

**Table 3:** Face-to-face follow-up appointment outcomes TTAD: telephone triage and advice

Year	2017	2018 (Including TTAD)	2018 (Excluding TTAD)
Total Number of Referrals	250	348	208
Treatment Completed	112 (44.8%)	91 (26.1%)	91 (43.8%)
Canceled Appointment	22 (8.8%)	19 (5.5%)	19 (9.1%)
Failed to Attend Appointment	40 (16.0%)	30 (8.6%)	30 (14.4%)
No Face-to-Face Follow-up Required	76 (30.4%)	208 (59.8%)	68 (32.7%)

Of the 140 patients discharged via the TTAD service, 41 were diagnosed with an ankle sprain, 31 with knee soft tissue injury (STI), 19 with shoulder STI, and 15 with lower back pain (LBP). The no-diagnosis group showed a higher rate of face-to-face appointments being offered when compared to being discharged via the service (Table 4).

**Table 4:** Outcome by the diagnosis of patients contacted via TTAD TTAD: telephone triage and advice, STI: soft tissue injury, LBP: lower back pain, OA: osteoarthritis

Diagnosis	Face-to-Face Appointment Made	Discharged via TTAD	Total
Ankle Sprain	18 (30.5%)	41 (69.5%)	59
Knee STI	11 (26.1%)	31 (73.8%)	42
Shoulder STI	14 (42.4%)	19 (57.6%)	33
LBP	10 (40.0%)	15 (60.0%)	25
Lower Quadrant Other	6 (46.2%)	7 (53.8%)	13
Neck STI	5 (45.5%)	6 (54.5%)	11
Foot STI	3 (37.5%)	5 (62.5%)	8
Upper Quadrant Other	3 (37.5%)	5 (62.5%)	8
OA Knee	3 (42.9%)	4 (57.3%)	7
Plantar Fasciitis	1 (20.0%)	4 (80.0%)	5
Elbow STI	0 (0.0%)	3 (100.0%)	3
Other	1 (100.0%)	0 (0.0%)	1
Total	75 (34.9%)	140 (65.1%)	215

## Discussion

The MSK physiotherapy outpatient services at Queens Hospital Burton observed an 11.2% increase in referrals received from the emergency department. This demonstrated an increase from 250 referrals made throughout August, September, and October in 2017 to 348 in 2018. The increase in referrals could be explained by the increasing number of attendances in the emergency department and the growing demand for MSK services [3,5]. Despite the increase in referrals to the MSK physiotherapy service, the number of initial face-to-face appointments reduced after the implementation of the TTAD service from 250 in 2017 to 208 in 2018. This demonstrated 16.8% fewer initial face-to-face appointments required despite the 11.2% increase in referrals. The pressure on initial face-to-face appointments was eased by discharging 140 patients via the TTAD service, that was 65.1% of the 215 patients that went through the TTAD service and 40.2% of the total number of referrals received from the emergency department that did not require an initial face-to-face appointment in 2018.

The effectiveness of the TTAD service was in part due to the type of MSK pathologies referred from the emergency department. The most common diagnosis seen via the TTAD service included ankle sprains (27.4%), knee STI (19.5%), shoulder STI (15.3%), and LBP (11.6%). The percentage of patients referred with a diagnosis of ankle sprain, knee STI, shoulder STI, and LBP that were discharged via the TTAD service was 69.5%, 73.8%, 57.6%, and 60.0% respectively.

These high percentages of discharge via the TTAD service can be explained by evidence relating to the management of these conditions. Advice and exercise therapy has been deemed to be effective in the management of ankle sprains, knee pain, and LBP [15]. Exercise therapy and advice have been demonstrated to be more effective in the management of acute ankle sprains when compared to electrotherapy or manual therapy [14]. Physiotherapy-led exercise and advice have been proven to be effective in the management of knee pain [16-18] with a single session of advice shown to be effective in the management of LBP [19,20]. Although these studies have not been proven in a virtual or remote clinical setting, it can be hypothesized that delivering exercise and advice via the phone can also be effective.

The impact of the TTAD service on failed attendance was also investigated. In 2017, there were 36 initial appointments and 40 follow-up appointments whereby patients failed to attend without prior communication. The number of failed attendances decreased in 2018 to 33 and 30 for initial and follow-up appointments respectively. The number of initial appointments not attended did not show a considerable difference between 2017 and 2018, but given the 11.2% rise in referrals, it may be considered effective in the fact the number of failed attendances did not also rise.

When comparing the number of failed attendances for initial appointments to the total number of referrals, there was a fall from 14.4% in 2017 to 9.5% in 2018. The high rate of failed attendances from referrals received from the emergency department could be in relation to the natural resolution of acute MSK complaints whilst waiting for the initial appointment, this may also be influenced by the initial exercises and advice offered by the emergency department in the form of handouts.

When comparing outcomes for follow-up appointments, the percentages did not differ between 2017 and 2018. A total of 174 patients in 2017 and 140 in 2018 required a face-to-face follow-up after the initial face-to-face appointment. The percentage of patients that completed physiotherapy treatment demonstrated a very slight increase in 2018 to 65.0% from 64.3% in 2017. Canceled follow-up appointments also illustrated a very slight rise from 12.6% in 2017 to 13.5% in 2018. The percentage of follow-up appointments with failed attendances did reduce in 2018 to 21.4% compared to 23.0% in 2017.

Although figures from 2018 differ from those in 2017, the changes are small and without a larger sample size, it is difficult to argue the significance of the TTAD service on follow-up appointments. This study only investigated the impact on the MSK physiotherapy service and therefore has not investigated the efficacy of TTAD nor the patients’ and physiotherapists’ satisfaction. Further research is required to investigate the clinical efficacy of a TTAD service in the initial management of patients referred to MSK physiotherapy services from the emergency department on a larger scale. Furthermore, secondary data should also assess the patient’s and physiotherapist’s satisfaction with running such a service.

## Conclusions

The introduction of a TTAD service for referrals from the emergency department has been demonstrated to be effective in reducing the number of face-to-face appointments required in the MSK physiotherapy management of these patients. Both initial and follow-up face-to-face appointments were lower in 2018 when compared to 2017, this is despite an 11.2% increase in the number of referrals throughout August, September, and October. Although there were only fewer failed attendances in 2018 when compared to 2017, that was despite an increase of 98 referrals from the emergency department. It can therefore be concluded that the TTAD service also had a positive impact on the failed attendance rate of initial face-to-face appointments.
